# Regional Variation in Restorative Treatment Need among Finnish Young People

**DOI:** 10.1155/2021/4852056

**Published:** 2021-11-10

**Authors:** Saujanya Karki, Antti Kämppi, Tarja Tanner, Jari Päkkilä, Marjo Seppänen, Leo Tjäderhane, Vuokko Anttonen, Pertti Patinen

**Affiliations:** ^1^Research Unit of Oral Health Sciences, University of Oulu, Oulu, Finland; ^2^Department of Oral and Maxillofacial Diseases, University of Helsinki, Helsinki, Finland; ^3^Medical Research Centre Oulu, Oulu University Hospital and University of Oulu, Oulu, Finland; ^4^Department of Mathematical Sciences, University of Oulu, Oulu, Finland; ^5^The Geography Research Unit, University of Oulu, Oulu, Finland; ^6^Oulu Deaconess Institute Foundation Sr., Department of Sports and Exercise Medicine, Oulu, Finland; ^7^Center for Life Course Health Research, University of Oulu, Oulu, Finland; ^8^Centre for Military Medicine, Finnish Defence Forces, Helsinki, Finland

## Abstract

**Aim:**

To evaluate the regional variation in restorative treatment need among Finnish young people based on the socioeconomic factors.

**Materials and Methods:**

This cross-sectional study was conducted in 20 garrisons of the Finnish Defence Forces in January and July 2011. The study population comprised 13,819 Finnish conscripts born in the beginning of 1990s, including females. A computer-based survey was done together with clinical oral examination to gather background information, e.g., educational status. Furthermore, average annual income of the conscript's residence municipality was achieved from the Statistics of Finland. The zip code of the place of residence of each conscript was later extracted from the Mildoc^®^ system. Georeferenced place of residence and income status were merged as information on provinces' level in a dataset. The association between the outcome variable and explanatory variables was determined by using the generalized linear mixed model, and geomaps were constructed.

**Results:**

Mean *D* value was 1.41 ranging from 0.89 (Kymenlaakso) to 2.33 (Kainuu). Higher education and high-income level were protective factors for restorative treatment need. Restorative treatment need was also low in those areas with high (OR: 0.70, 95% CI: 0.56–0.87) and medium (OR: 0.79, 95% CI: 0.70–0.89) yearly income compared to low yearly income. The high odds for the need of restorative treatment were discovered in Northern Ostrobothnia (OR: 2.26, 95% CI: 1.53–3.33) followed by Central Ostrobothnia (OR: 2.08, 95% CI: 1.17–3.70), Uusimaa (OR: 1.55, 95% CI: 1.16–2.08), and Central Finland (OR: 1.54, 95% CI: 1.10–2.16) compared to Varsinais-Suomi.

**Conclusion:**

In conclusion, there is a significant regional variation in restorative treatment need among Finnish young people in their twenties based on the socioeconomic factors.

## 1. Introduction

In Finland, municipalities are responsible for organizing the primary health services including oral health services. Children (until 18 years) receive all health services including oral health free of charge. Similarly, after the major reform in 2001-2002, adults (>18 years) are entitled to access the public dental service (PDS) as well as subsidized basic services in private dental services [1]. The fund for PDS is mostly covered by the taxation generated with the municipalities and partly paid by the central government. However, there is evidence of polarization of dental caries among young [2] and middle-aged adults [3]. Likewise, the socioeconomic inequalities in dental caries status are still persistent among Finns [4, 5].

The use of spatial analyses, particularly geomapping, is increasing in public health research. Geomapping helps to evaluate the geographical variations of health outcomes with respect to risk factors such as demographic measures, socioeconomic inequalities, or behavioural factors and illustrates the spatial distribution of health outcomes [6]. In the field of dental public health, geomapping has been used in dental diseases' surveillance, evaluating the geographical variation of dental diseases, utilization of oral health services, and dental workforce planning [7].

In a previous study, Kämppi et al. showed that location (urban) and environment (high level of fluoride content in drinking water and living in Swedish-speaking area) were associated with dental caries experience [8]. In the same study, cartograms were drawn to illustrate the mean restorative treatment needs among young adults using the Geographic Information System (GIS).

According to the Statistics of Finland, there is a variation of education level, average annual income, and consumption of oral health services within the country [9]. However, the association between restorative treatment needs and average annual municipal income among Finnish young people is not known. Therefore, this study aims to investigate the regional variation in restorative treatment need among Finnish young people based on the socioeconomic factors. The study hypothesis was that area with low average annual income is associated with the high caries experience.

## 2. Materials and Methods

This cross-sectional study was conducted in 20 garrisons of the Finnish Defence Forces in January and July 2011. The study population comprised conscripts born in the beginning of 1990s. A total of 13,819 conscripts including females (*n* = 255), born in 1990, 1991, or 1992, participated in this study. A computer-based survey was done together with clinical oral examination to gather background information, e.g., educational status.

### 2.1. Clinical Oral Examination

Clinical oral examination was performed by 15 military dentists according to the Defence Forces protocol. Restorative treatment need was recorded per tooth at the dentin caries level (DT). The inter- and intraexaminer agreement were substantial (interexaminer agreement was 0.71, and intraexaminer agreement was 0.72). Details of the study were explained previously [8].

### 2.2. Geographical Information

The zip code of the place of residence of each conscript was later extracted from the Mildoc^®^ system. Income status of the municipality for the year 2011 was achieved from the Statistics of Finland [9]. Later, georeferenced place of residence and income status were merged as information on provinces in a dataset.

### 2.3. Statistical Considerations

For analyses, the mean DT values were calculated and categorized as very good (0.91–1.27), good (1.28–1.64), poor (1.65–2.00), and very poor (2.01–2.36) for each municipality as described by Kämppi et al. [8].

The association between the outcome variable and explanatory variables was determined by using the generalized linear mixed model. In the mixed model, garrison was taken into consideration as the random effect. For analyses, the outcome variable, restorative treatment need, was dichotomized as DT = 0 and DT ≥ 1, and the explanatory variables were conscripts' own educational status (vocational school, matriculation exam or higher secondary school, and others), average municipal annual income (based on quartiles as the 1st quartile (<17000€), 2nd quartile (17000€–19449€), 3rd quartile (19450€–21750€), and 4th quartile (>21750€)), and place of residence (province). The association between dental caries experience (DT = 0 and DT ≥ 1) and place of residence was adjusted with average municipal annual income and educational background in the model and presented in geomaps. Geomaps were constructed using ArcGIS^®^ Pro 2.6.2 software (Esri, Redlands, California). Odds ratios (OR) with 95% confidence interval (95% CI) were presented in geomaps. For all analyses, *p* < 0.05 was considered statistically significant. All analyses were executed using R software version 4.0.2 (R Core Team, Vienna, Austria).

### 2.4. Ethical Issues

The research plan was accepted by the Ethics Committee of Northern Ostrobothnia Hospital District on March 29, 2010. The Center for Military Medicine and the Defence Staff gave the permission for the study in June 2010 (AG14218/June 23, 2010). For the analyses, the IDs were excluded.

## 3. Results

Educational background of the study participants, average annual income of the municipality they reside, and the mean dental caries in the province level are presented in Table 1.

Caries experience was more common among those who had annual average income <17000€ and had vocational or other education. Higher education and high-income level in the province of residence were protective factors for restorative treatment need. Restorative treatment need was low in those areas with average annual income in the 3rd quartile (OR: 0.83, 95% CI: 0.68–1.00) and 4th quartile (OR: 0.87, 95% CI: 0.71–1.06) compared to those areas with average annual income in the 1st quartile (Table 2).

**Figure 1:**
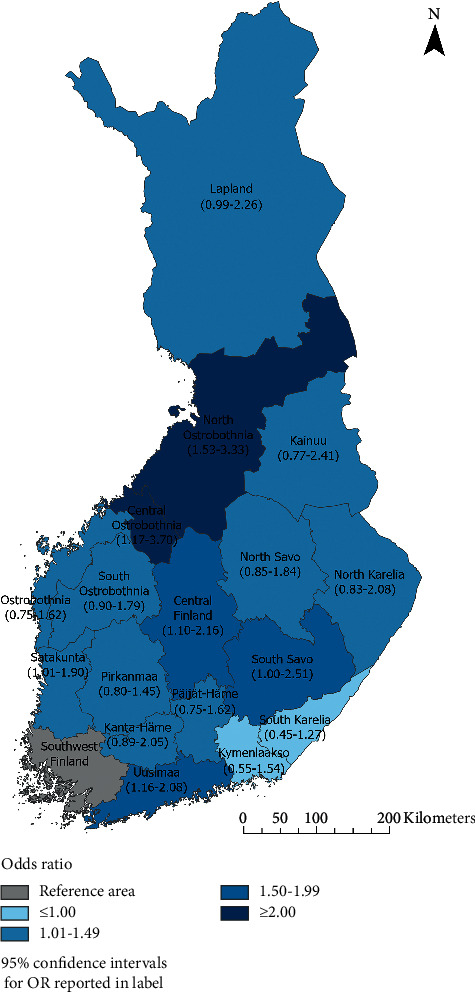
Geomap presenting the association between dental caries experience (DT > 0 vs. DT ≥ 1) and the place of residence among the Finnish conscripts.

In the regression model adjusted with average annual municipal income and educational background, the most highest odds for the need of restorative treatment were discovered in Northern Ostrobothnia (OR: 2.26, 95% CI: 1.53–3.33) followed by Central Ostrobothnia (OR: 2.08, 95% CI: 1.17–3.70), Uusimaa (OR: 1.55, 95% CI: 1.16–2.08), and Central Finland (OR: 1.54, 95% CI: 1.10–2.16) compared to Varsinais-Suomi (Figure 1).

## 4. Discussion

This cross-sectional study aimed to evaluate the regional variation in restorative treatment need among Finnish young people based on the socioeconomic factors. Restorative treatment need was low among those living in the municipality with higher annual income compared to those areas with low average annual income. In addition, regional difference was found to be associated with dental caries occurrence in this study.

One of the strengths of this study is the large number of the cohort born in the beginning of 1990s including females. Another is high participation rate due to obligatory oral health examination of the conscripts. Furthermore, easy interpretation of the geomapping gives an overall view of inequalities related to oral health. The cross-sectional nature can be considered as the limitation of this study.

Suominen et al. reported an improvement in the mean number of teeth with dental caries among Finnish adults over a period of 11 years [10]. However, an alarming situation with respect to enamel caries was reported in a recent study conducted among the participants of Northern Finland Birth Cohort 1966 [3]. Previously, a systematic review reported that the low socioeconomic status is associated with high dental caries experience, and this association is more pronounced in high-income countries [11]. Similarly, a recent study also concluded that the low level of education was associated with a higher occurrence of dental caries as well as periodontal disease [5]. These findings are in concordance with the present study.

Numerous epidemiological studies have shown that the differences between the areas concerning both dental health and attendance are considerable. Respondents living in Eastern Finland had lost more teeth and visited a dentist more seldom than those living in the southern parts of the country [12]. In Finland, a dental subsidization reform, implemented in 2001-2002, abolished age restrictions on subsidized dental care. However, in recent studies, the income-related inequalities in perceived oral health have remained or even widened after the reform [13].

According to results based on the Finnish Institute for Health and Welfare (THL), regional differences are observed in disease morbidity and mortality. In Eastern Finland, morbidity and mortality have traditionally been higher than average when compared to Western Finland [14]. This phenomenon with respect to dental caries experience is also clearly seen in the results of the present study. An interesting finding, however, is the poor results of caries experience in two western counties, namely, Central Ostrobothnia and Satakunta regions, compared to Southwest Finland. In both counties, there are large Swedish-speaking communities. In previous studies, Swedish as the spoken mother language has been considered as a protective factor against dental caries [8]. Obviously, further studies are needed to clarify these contractionary findings.

Because of the obligatory military service for all males in Finland, there is a great opportunity to carry out epidemiological studies on oral and general health of the young people. In addition, female conscripts who underwent military service voluntarily were also included in this study. Although the search methods on indexes and databases have improved during the past years, the nationwide epidemiological studies concerning dental caries are sparce. The material for the present study was collected during the first service week of the conscripts thus presenting the epidemiological status of diseases of a whole age group in Finland. Furthermore, the garrisons were taken into consideration as the random effect in the mixed model for generalizability.

## 5. Conclusion

Geomapping based on the home addresses of the patients provides an easy and cost-effective method to study the areal epidemiology of diseases. In conclusion, there is a significant regional variation in restorative treatment need among Finnish young people in their twenties based on the socioeconomic factors.

## Figures and Tables

**Table 1:** Distribution of study participants based on their education background and the average annual income of the municipality and mean tooth decay in the province level.

Province	Education				Average yearly income					Mean decay
	Vocational	Matriculation or higher secondary	Others	Total	<17000€	17000€–19449€	19450€–21750€	>21750€	Total	
Central Finland	47.5	42.2	9.4	100.0	11.5	11.9	67.9	8.6	100.0	1.58
Central Ostrobothnia	57.3	36.0	6.7	100.0	8.7	9.6	81.7	0.0	100.0	2.17
Kainuu	44.2	48.1	7.8	100.0	41.1	0.0	58.9	0.0	100.0	2.33
Kanta-Häme	48.4	42.2	9.4	100.0	0.0	11.6	4.6	83.8	100.0	1.64
Kymenlaakso	57.1	33.9	8.9	100.0	0.0	0.5	14.6	84.8	100.0	0.89
Lapland	52.8	35.1	12.1	100.0	12.5	12.5	69.4	5.5	100.0	1.31
North Karelia	51.1	39.4	9.5	100.0	21.2	64.6	5.8	8.5	100.0	1.36
North Ostrobothnia	43.1	47.3	9.5	100.0	7.5	13.2	18.9	60.4	100.0	1.71
North Savo	53.9	38.5	7.6	100.0	8.2	12.7	66.1	12.9	100.0	1.52
Ostrobothnia	43.9	49.5	6.5	100.0	0.0	11.9	49.3	38.8	100.0	0.97
Pirkanmaa	45.3	44.6	10.1	100.0	1.2	11.9	57.2	29.6	100.0	1.57
Päijät-Häme	40.7	43.1	16.2	100.0	6.3	0.0	84.4	9.3	100.0	1.45
Satakunta	50.8	39.4	9.7	100.0	6.5	13.8	52.6	27.2	100.0	1.86
South Karelia	50.5	36.8	12.6	100.0	13.3	6.7	77.3	2.7	100.0	1.31
South Ostrobothnia	53.3	39.8	6.9	100.0	12.0	56.9	10.5	20.6	100.0	1.69
South Savo	45.8	42.8	11.4	100.0	17.4	35.0	47.7	0.0	100.0	1.26
Southwest Finland	45.8	41.1	13.1	100.0	0.0	8.9	60.6	30.5	100.0	1.12
Uusimaa	29.4	56.3	14.3	100.0	0.0	0.0	0.9	99.1	100.0	1.21

**Table 2:** Association between dental caries experience (DT > 0 vs. DT ≥ 1) and educational background and average municipal income among the Finnish conscripts.

Explanatory variables	OR	95% CI
*Educational background*		
Vocational	1	
Matriculation or higher secondary	0.5	0.45–0.55
Others	1.6	1.40–1.90

*Average municipal annual income*		
<17000€	1	
17000€–19449€	1.1	0.86–1.31
19450€–21750€	0.8	0.68–1.00
>21750€	0.9	0.71–1.06

## Data Availability

The data presented in this study are available from the corresponding author upon request. The data are not publicly available due to ethical restrictions.
